# Secondary malignancy of T-cell origin after CAR T-cell therapy: EMA’s conclusions from the evaluation of 38 suspected cases

**DOI:** 10.1038/s41434-025-00586-x

**Published:** 2025-12-22

**Authors:** Philipp Berg, Charlotte Bakker, Moritz Sander, Nicklas Hasselblad Lundstrøm, Karin Erneholm, Flora Musuamba Tshinanu, Olga Kholmanshikh, Filip Van Nuffel, Susanne Müller, Gabriele Ruppert-Seipp, Gabriele D. Maurer, Justina Januskiene, Maria Mantziri, Bianca Mulder, Frederika A. van Nimwegen, Daiana Vasilcanu, Ulla Wändel Liminga

**Affiliations:** 1 Safety of Biomedicines and Diagnostics, Paul-Ehrlich-Institut, Langen, Germany; 2 Quality and Safety of Medicines Department, European Medicines Agency (EMA), Amsterdam, The Netherlands; 3 The Danish Medicines Agency, Copenhagen, Denmark; 4 Pharmacovigilance Risk Assessment Committee (PRAC), European Medicines Agency (EMA), Amsterdam, The Netherlands; 5 Federal Agency for Medicines and Health Products, Brussels, Belgium; 6 Medicines Evaluation Board, Utrecht, The Netherlands; 7 Swedish Medical Products Agency, Uppsala, Sweden

**Keywords:** T-cell lymphoma, Myeloma

## Abstract

This article provides a regulatory perspective on secondary malignancy of T-cell origin as a rare adverse reaction to the currently marketed CD19- or BCMA-directed chimeric antigen receptor (CAR) T-cell therapies. To assess the risk, causality between reported suspected adverse reactions and CAR T-cell therapy was assessed applying the principles of the World Health Organization-Uppsala Monitoring Centre causality categories, alongside a review of scientific publications and data from registries/ databases. By 11 April 2024, 38 cases of T-cell malignancy after CAR T-cell therapy were reported in patients aged 29–80 years. In 19 patients, tumour samples were tested for the presence of CAR transgene, which was detected in seven cases. Most of the T-cell malignancies were diagnosed within 12 months of treatment (22/33; 67%). The reporting rate is approximately one case per 1000 patients treated. An overall causal relationship was established with at least a reasonable possibility. Regulatory measures included updates to the product information, risk management plan, and educational materials. An additional pharmacovigilance activity was requested from the marketing authorisation holders (MAHs) to strengthen the process of genetic testing of residual tumour samples. To further characterise this risk and understand underlying mechanisms, continued efforts from healthcare professionals, MAHs and regulators are essential. Well-documented case reports, including information on genetic testing of tumour samples, are considered crucial elements.

## Introduction

Chimeric antigen receptor (CAR) T-cells are T-cells that have been genetically modified to express a CAR. Between 2018 and 2024, the European Medicines Agency (EMA) issued positive opinions for the approval of six CAR T-cell products indicated for the treatment of specific types of relapsed or refractory B-cell malignancies. These products are tisagenlecleucel (Kymriah), lisocabtagene maraleucel (Breyanzi), axicabtagene ciloleucel (Yescarta), brexucabtagene autoleucel (Tecartus), idecabtagene vicleucel (Abecma) and ciltacabtagene autoleucel (Carvykti), which are either cluster of differentiation 19 (CD19)- or B-cell maturation antigen (BCMA)-directed. New CAR T-cell products are currently in development or under assessment for marketing authorisation, including those targeting non-malignant conditions, such as autoimmune diseases [1–3].

The currently approved products use non-replicating lentiviral or γ-retroviral vectors to integrate the genetic material into the genome of autologous T-cells. The vectors were designed to minimise the risk of insertional mutagenesis, but it remained at least a theoretical concern [4]. Because vector insertion may affect the expression of genes that play a role in cell proliferation, differentiation or survival, it could contribute to oncogenesis [5–7]. Therefore, the development of a secondary malignancy was considered a potential risk for CAR T-cell products at the time of marketing authorisation. This potential risk is described in the product information, which includes a recommendation to monitor patients life-long. Additionally, a product-specific regulatory document (the risk management plan) specifies that for each product, this risk has to be closely monitored through pharmacovigilance activities. These activities include the evaluation of new safety data in periodic safety update reports, submitted by the responsible marketing authorisation holder (MAH) for review by the European medicines regulatory network at agreed intervals, and the conduct of post-authorisation safety studies. Furthermore, regular monitoring of spontaneously reported cases of suspected adverse reactions, as well as of literature within signal detection activities, is undertaken by the MAH and the EU regulatory system.

At the end of 2023, multiple cases of T-cell malignancy after CD19- or BCMA-directed CAR T-cell therapy had been reported as suspected adverse reactions and communicated by the United States Food and Drug Administration (FDA) [8]. This prompted an in-depth review of available data by EMA. Some of these and other individual case reports of patients developing a T-cell malignancy after receiving CAR T-cell therapy have been published [9–14].

The current article provides a high-level summary of the 38 cases reporting T-cell malignancy following CD19- or BCMA-directed CAR T-cell therapy identified and evaluated by EMA. We describe how this risk was assessed, including the assessment of causality between the development of T-cell malignancy and the CAR T-cell products, and discuss the interplay needed between clinicians, MAHs and regulators to gain further insight into secondary malignancies of T-cell origin following CAR T-cell therapy.

## Methods

### Data collection

As part of EMA’s signal management process [15], cases of secondary malignancy of T-cell origin reported with any of the six approved CAR T-cell products were extracted from EudraVigilance. EudraVigilance is a system for collecting, managing and analysing suspected adverse reactions to medicines authorised in the European Economic Area (EEA) [16]. Cases were identified using Medical Dictionary for Regulatory Activities (MedDRA) version 26.1 high-level group term Lymphomas non-Hodgkin’s T-cell; high-level term Leukaemias chronic T-cell, preferred terms (PTs) T-cell type acute leukaemia, Lymphoproliferative disorder, and Lymphocytic leukaemia.

This summary of cases prompted a formal signal evaluation procedure led by EMA’s Pharmacovigilance Risk Assessment Committee (PRAC), which started by requesting the MAHs to submit cumulative reviews of secondary T-cell malignancy cases, which had been received from all sources, including clinical trials, observational studies and spontaneous reports, for their respective product. The MAHs were also requested to provide exposure data (number of treated patients) for their respective product. To further assess the potential risk of developing a T-cell malignancy after CAR T-cell therapy, background incidence rates of T-cell malignancies in the patient populations eligible for CAR T-cell therapy were requested. Furthermore, MAHs were asked to discuss potential mechanisms underlying the development of T-cell malignancies following CAR T-cell therapy, as well as the need for any regulatory action, such as updating the product information. Any new information that became available during the regulatory procedure up to 11 April 2024 was included in the final review.

### Causality assessment

To characterise the risk, the potential causal relationship between secondary malignancy of T-cell origin and the respective CAR T-cell product was assessed according to the principles of the World Health Organization-Uppsala Monitoring Centre (WHO-UMC) causality categories for each case [17]. The WHO-UMC system is a tool to aid causality assessment of suspected adverse reactions, with the aim of determining the level of plausibility of a causal relationship between an event and a medicinal product [17]. This tool is intended to classify the likelihood of such relationship in a structured, standardised approach.

The WHO-UMC method to assess causality was adapted to increase applicability due to the nature of these products and the suspected adverse reactions. As a CAR T-cell product is generally administered once, the established concept of assessing the effect of discontinuation (dechallenge) and reintroduction (rechallenge) of the treatment on an adverse event was not applicable. Also, no dose-effect relationship can be expected, as may be the case for other medicines. On the other hand, tumour samples can be tested for the presence of the CAR transgene and its integration sites, thus a modified assessment algorithm with molecular analysis as a crucial element has been proposed [18].

The causality was considered “probable” in cases where a tumour sample consisted predominantly of a transgene-containing clone, in particular if the vector insertion(s) occurred in specific genomic regions that could affect cell proliferation or fate. It was considered sufficiently plausible that the CAR T-cell product had contributed to the oncogenesis. The causality was established “possible” for reports of newly diagnosed T-cell malignancy occurring after CAR T-cell therapy when information on testing of tumour material was not available. The neoplasm could have been caused or promoted by an insertional mutagenesis event or have developed for other reasons. Of note, causality was not by default considered “possible”, if there were more likely explanations for the T-cell malignancy. If genetic testing of a tumour sample was performed but did not identify transduced (transgene-containing) cells, insertional mutagenesis and subsequently causality was considered “unlikely” and an alternative cause was considered a more plausible explanation, including treatment-independent factors. There are various risk factors that can increase the susceptibility of an individual to develop a secondary malignancy (e.g., predisposing germline mutation; prior chemotherapy). However, in most cases, the actual contribution of such factors towards the manifestation of a secondary T-cell malignancy cannot be quantified and was therefore not considered as strong evidence against an impact of the CAR T-cell therapy. Overall, it should also be noted that these causality assessments cannot and are not intended to prove definitive causality between a condition and a treatment in an individual case.

## Results

### Summary of the cases

**Fig. 1:**
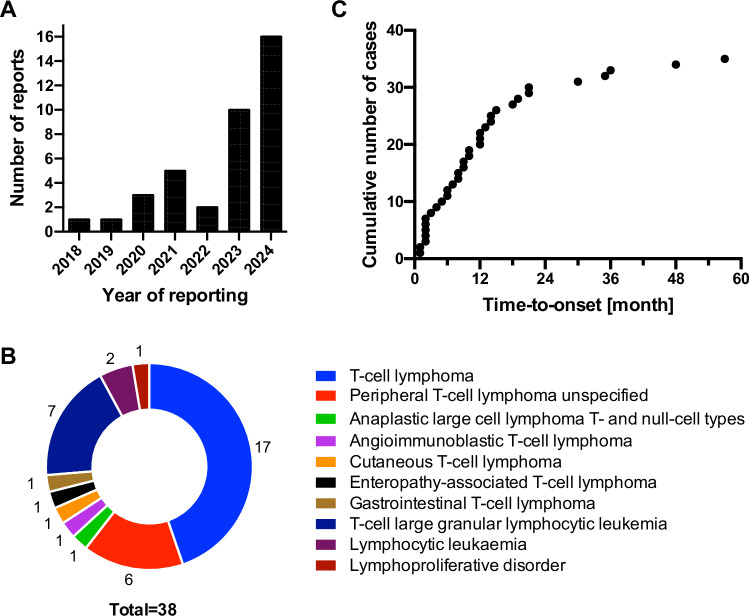
Characterisation of reported secondary malignancies of T-cell origin after CAR T-cell therapy. **A** Number of secondary malignancies of T-cell origin following CAR T-cell therapy reported per year up to 11 April 2024. **B** MedDRA preferred terms reported for malignancies of T-cell origin. **C** Time-to-onset (TTO) for malignancies of T-cell origin after CAR T-cell therapy in months. TTO was unknown in three out of 38 cases.

Up to 11 April 2024, in total, 38 cases of T-cell malignancy following CAR T-cell therapy had been reported for five of the six approved CAR T-cell products. Six cases were reported from the clinical trial setting and 32 cases were reported from post-marketing sources. Reports concerned 15 males, 17 females, and 6 individuals with unknown sex. For 26 cases, the patients’ age was reported (median: 60 years; range: 29–80 years). The majority of cases originated from the United States (*n* = 25), and six cases were reported within the EEA. Thirty seven cases were reported by healthcare professionals, one case was derived from literature. The number of reported cases increased since the end of 2023 (Fig. 1A).

Different MedDRA PTs relating to T-cell malignancies were reported, the most frequent one being T-cell lymphoma (*n* = 17) (Fig. 1B). For these 17 cases, the type of T-cell lymphoma was not further specified. Many of the identified T-cell malignancies had been diagnosed within the first 12 months post CAR T-cell therapy (22/35; 63%) (Fig. 1C). For half of all cases (19/38), no tumour samples had been tested for the presence of the CAR transgene, while for the other 19 cases, analyses had been undertaken using different methodologies. Reasons for not testing included absence of suitable samples or lack of patient consent, but in many cases, it was not reported why relevant molecular testing had not been undertaken.

Of the 19 tumour samples tested, seven consisted of CAR-positive tumour cells. No transcriptome data were available for any of the cases with CAR-dominant clones at the time of assessment. A total of seven samples were negative for the transgene, which indicates T-cell transformation independent of vector insertion. In five cases, the tumour samples contained a low copy number of the transgene, which was hypothesised to be due to CAR T-cells infiltrating the tumour tissue and was not considered to be suggestive of a CAR T-cell-derived dominant cell population.

For the time being, no replication-competent lentivirus (RCL) or y-retrovirus (RCR) has been detected at any available time point for all cases with available test results.

Causality was considered ‘probable’ in seven cases based on the presence of the CAR transgene in tested samples. In 13 cases, causality was assessed as ‘possible’ in the absence of CAR transgene testing. In twelve cases, causality was categorised as ‘unlikely’, following negative or insignificant CAR transgene test results, which indicated that no vector insertion had taken place in the tumour-forming cells, a prerequisite for insertional mutagenesis. In five cases, causality was not assessable due to inconclusive or contradictory information. In one case, causality was classified as conditional, pending results of transgene testing. The submitted data did not indicate any obvious differences between the products. See Table 1 for an overview of the case characteristics based on the CAR target and used vector.

**Table 1:** Overview of case characteristics, tumour sample test results, and causality assessment.

CAR T-cell therapy	Age (years)	Sex	Time to onset (months)	MedDRA PT classification	Tumour sample tested	CAR transgene test result	Causality assessment
BCMA directed LVV *N* = 9 cases	47–71 1 case unknown	4 Female 4 Male 1 Unknown	2–21	• Anaplastic T-cell lymphoma *n* = 1 • Enteropathy-associated T cell lymphoma *n* = 1 • Gastrointestinal T-Cell Lymphoma *n* = 1 • Large granular lymphocyte (LGL) leukaemia *n* = 1 • Peripheral T-Cell Lymphoma *n* = 3 • T-cell lymphoma *n* = 2	Yes *n* = 7 No *n* = 2	Positive *n* = 5 Negative *n* = 2	Probable: *n* = 5 Unlikely *n* = 2 Conditional *n* = 1 Not assessable *n* = 1
CD19-directed LVV *N* = 13 cases	46–75 5 cases unknown	6 Female 5 Male 2 Unknown	1–35 2 cases unknown	• Peripheral T-Cell Lymphoma *n* = 3 • Cutaneous T-cell lymphoma *n* = 1 • Large granular lymphocyte (LGL) leukaemia *n* = 1 • T-cell lymphoma *n* = 8	Yes *n* = 8 No *n* = 5	Positive *n* = 2 Negative *n* = 6	Probable *n* = 2 Possible *n* = 5 Unlikely *n* = 6
CD19-directed γ-RVV *N* = 16 cases	29–64 6 cases unknown	7 Female 7 Male 2 Unknown	2–57 1 case unknown	• Angioimmunoblastic T-cell lymphoma *n* = 1 • Lymphoproliferative Disorder *n* = 1 • Large granular lymphocyte (LGL) leukaemia *n* = 5 • Lymphocytic leukaemia *n* = 2 • T-cell lymphoma *n* = 7	Yes *n* = 4 No *n* = 12	Negative *n* = 4	Possible *n* = 8 Unlikely *n* = 4 Not assessable *n* = 4

*BCMA directed LVV* B-cell maturation antigen directed lentiviral vector (i.e., idecabtagene vicleucel and ciltacabtagene autoleucel), *CD19-directed LVV* cluster of differentiation 19 directed lentiviral vector (i.e., lisocabtagen maraleucel, tisagenlecleucel), *CD19-directed RVV* cluster of differentiation 19 directed γ-retroviral vector (i.e., axicabtagene ciloleucel, brexucabtagene autoleucel).

### Magnitude of the risk

By 11 April 2024, ~42,500 patients had received one of the authorised CAR T-cell therapies in clinical trials or in the commercial setting. With 38 reported cases of T-cell malignancy following CAR T-cell therapy, the reporting rate is approximately one case per 1000 patients treated.

To put the number of cases observed into context, further understanding of the background incidence of T-cell lymphoma among patients with B-cell malignancies was sought from the literature. Patients with B-cell malignancies are at an increased risk of developing a subsequent T-cell malignancy compared with the general population; Chihara et al. reported that patients with B-cell lymphoma have an almost five-fold increased risk of T-cell lymphoma [19]. Dores et al. found a similar relative risk and estimated an excess absolute risk of 18 cases per 100,000 person-years [20]. Both studies estimated a cumulative incidence of secondary T-cell neoplasms of <0.15% [19, 20].

### Regulatory actions

Following thorough analysis, PRAC concluded that there is a risk of developing a T-cell malignancy after CD19- or BCMA-directed CAR T-cell therapy. The product information, the risk management plan and the educational material for healthcare professionals are being updated to reflect that secondary malignancy of T-cell origin is a known risk for these therapies. Further, a letter was distributed to relevant healthcare professionals in the EU, following a defined communication plan, to inform about the findings and raise awareness for patient monitoring. The PRAC required an additional pharmacovigilance activity from the respective MAHs if not yet in place; MAHs need to have an appropriate framework and process guidance in place to support and facilitate the collection and genetic testing of residual tumour samples from patients who have developed a secondary malignancy of T-cell origin in the post-marketing setting.

## Discussion

After a thorough evaluation of available data on secondary T-cell malignancies following CD19- or BCMA-directed CAR T-cell therapy, including 38 patient cases, EMA concluded that it is at least a reasonable possibility that malignancies of T-cell origin are causally related to these therapies. Based on the available data, and particularly taking the mechanisms and effects of these products into account, EMA concluded that it is a class effect, even if not all products had reported cases of T-cell malignancy by 11 April 2024. This picture is not unexpected, given the estimated rarity of the event and the relatively few subjects being treated with some of the products, and also taking the relatively limited follow-up time for most of the patients into account. EMA’s conclusions led to regulatory actions focused on risk minimisation measures. The benefit/risk balance for the approved CAR T-cell products remains positive, given the rarity of the event and the efficacy shown for all products in their respective indications. It is noted that the number of cases reported by the end of 2023 (*n* = 25) increased during the review (*n* = 38 by 11 April 2024). One reason may be increased awareness following the FDA’s announcement in November 2023 of an investigation of this event, as well as subsequent publications in high-impact scientific journals [21]. Further, from end of 2023, the estimated exposure increased by several thousand patients, and additional case reports were therefore expected during the review.

Overall, the risk of secondary malignancy of T-cell origin seems low, as the reporting rate is about one case among 1000 patients treated with CD19- or BCMA-directed CAR T-cell therapies, based on current evidence from spontaneously reported cases. While patient exposure is considered unusually certain in this case, since patients are given one dose only, and for each patient, a unique dose is produced – the reporting rate might still be an underestimation as underreporting is a well-known limitation in post-marketing safety monitoring [22]. It should be noted that the reporting rate indicates the number of cases reported in relation to the number of patients treated, irrespective of the outcome of the causality assessment of a particular case. There are several recent studies describing the reporting rates of secondary T-cell malignancy following CAR T-cell therapy, namely Hamilton et al. [23] reporting one case among 724 patients, Ghilardi et al. [13] reporting one case among 449 patients, Levine et al. [24] reporting three cases among 11,345 patients, Tix et al. reporting five cases among 5517 patients, and Lorenc et al. [25] reporting no cases among 355 patients [13, 23–26]. Currently available epidemiological data on the risk of T-cell malignancy following CAR T-cell therapy is limited due to the low reporting rate, the relatively small number of patients being observed and the currently still restricted follow-up time.

In the studies reporting the background incidence of secondary T-cell malignancy among patients not treated with CAR T-cell therapy and corresponding to approved indications, the development of T-cell neoplasms varied substantially according to the subtype of the underlying malignancy across analyses [19, 20]. Data on the background risk in multiple myeloma and acute lymphocytic leukaemia populations were very limited. Based on the low reporting rates, and in the absence of suitable comparator populations unexposed to CAR T-cell therapies but with similar baseline characteristics, estimations of the relative risk of T-cell malignancies could not be ascertained.

Reporting of suspected adverse reactions by healthcare professionals or patients to regulatory agencies and MAHs is a cornerstone in post-marketing safety monitoring. The analysis of case reports provides valuable safety information on the products used in clinical practice and, therefore, can complement information from clinical trials. Clinical trials are usually conducted in populations defined by strict inclusion and exclusion criteria, involve a limited number of subjects, and can therefore only identify more common adverse reactions. Healthcare professionals have a duty to report suspected adverse reactions, but underreporting is common in post-marketing safety monitoring [22]. Several factors contribute to underreporting, including a lack of time to complete reporting forms and uncertainty about the causal relationship between medicine and the event [27]. However, it should be noted that assessment of causality is not needed for reporting a suspected adverse reaction. Overall, the value of reporting suspected adverse reactions is exemplified by the review described in this article, which hopefully motivates healthcare professionals to report suspected adverse reactions in the future.

Assessing causality in this procedure was challenging, partly since some of the factors generally considered in causality assessments, such as dechallenge, rechallenge or dose-effect relationships, were not applicable. Detailed documentation of patient characteristics including medical history, previous treatments with potentially contributing effect (e.g., chemotherapy), details on the diagnosis of the T-cell malignancy, and in particular test results for the presence of CAR transgene in the tumour tissue were crucial for enabling a thorough causality assessment and are desirable elements to be included in future cases being reported in the post-marketing setting.

Although testing for the transgene was lacking in half of the reviewed cases, the available data allowed PRAC to conclude that, with a reasonable possibility, there is a causal relationship between treatment with CAR T-cells and development of a secondary T-cell malignancy. To further understand underlying mechanisms and consequently the relationship between anti-CD19 or anti-BCMA CAR T-cell products and the development of T-cell malignancy, information on transgene expression, clonality of the cell population, vector integration site(s) and transcriptome analysis would be needed [18].

However, genetic testing of tumour samples for the presence of the CAR vector is not a routine procedure. In most cases, such analyses have no consequences for the prognosis or treatment choice for the individual patient. Therefore, testing needs to be undertaken within a defined research context. Collecting and processing residual tumour samples require patients’ informed consent and approval by local regulations including ethics committee. Consequently, EMA required MAHs to have an appropriate framework in place, as described in the results section. As recommended in the product information for the CAR T-cell products, healthcare professionals should contact the MAHs to obtain instructions for the testing of the tumour samples.

In addition to reviewing spontaneously reported post-marketing cases, MAHs are conducting post-authorisation safety studies to further characterise, among other objectives, the risk of secondary T-cell malignancy. These ongoing studies utilise mainly data from Center for International Blood and Marrow Transplant Research (CIBMTR) and the European Society for Blood and Marrow Transplantation (EBMT) registries. The aim is to ensure 15 years of follow-up to further delineate the long-term safety profile of these products. It is therefore important that patients are encouraged to enrol for registry follow-up.

Underlying mechanisms for the development of secondary malignancy of T-cell origin in CAR T-cell-treated patients have not been elucidated. Various aspects can be considered. In addition to the risk of insertional mutagenesis, non-mutational changes during the manufacturing process could affect the regulation of gene expression, contribute to clonal expansion and influence cell fate, although there is no clear evidence up to now [28–30]. Besides, patients receive lymphodepleting chemotherapy prior to treatment with CAR T-cell products, which may also attenuate their capacity to eliminate premalignant cells. Genetic predispositions and clonal haematopoiesis may render patients receiving these products more susceptible to developing malignancies. Indeed, cases of CAR-negative T-cell malignancies with pre-existing clonal haematopoiesis mutations have been reported, and it was speculated that CAR-mediated inflammation may drive the expansion of such clones and contribute to T-cell tumorigenesis [23, 31]. Regardless of insertional mutagenesis, the accumulation of predisposing therapies, the immune dysregulation along with the T-cell activation during manufacturing and the subsequent inflammation may result in tumorigenesis via a multifactorial mechanism [32].

## Conclusion

By 11 April 2024, in total 38 cases of secondary malignancy of T-cell origin following BCMA- or CD19-directed CAR T-cell therapy were identified. In seven of these (18%), causality between the CAR T-cell therapy and secondary T-cell malignancy was considered probable mainly based on detection of the CAR transgene in the tumour cells. Overall, causality between these types of CAR T-cell therapies and occurrence of secondary malignancy T-cell origin is established with at least a reasonable possibility. The product information for these therapies has been updated to reflect these data. Secondary T-cell malignancy is recognised as a rare adverse reaction. Nevertheless, healthcare professionals and patients should be aware of this risk. The benefit/risk balance for the approved CAR T-cell products remains positive.

Further actions from healthcare professionals, MAHs and regulators are required to better understand the underlying mechanisms, to further characterise and possibly minimise this risk. Well-documented case reports and results from genetic testing are desirable and a prerequisite for a comprehensive assessment. The framework and process guidance as required by EMA are intended to further support clinicians and patients in the process to allow testing of residual tumour samples, and thereby generate more data that can help in further evaluating underlying mechanisms.

## Data Availability

The datasets generated during and/or analysed during the described procedure are not publicly available due to legal restrictions.
